# Identification and validation of a miRNA-based prognostic signature for cervical cancer through an integrated bioinformatics approach

**DOI:** 10.1038/s41598-020-79337-4

**Published:** 2020-12-17

**Authors:** Yumei Qi, Yo-Liang Lai, Pei-Chun Shen, Fang-Hsin Chen, Li-Jie Lin, Heng-Hsiung Wu, Pei-Hua Peng, Kai-Wen Hsu, Wei-Chung Cheng

**Affiliations:** 1 Department of Obstetrics and Gynecology, Suzhou BenQ Medical Center, The Affiliated BenQ Hospital, Nanjing Medical Medical University, Suzhou, 215010 Jiangsu China; 2 Graduate Institute of Biomedical Science, China Medical University, Taichung, 40403 Taiwan, ROC; 3 Department of Radiation Oncology, China Medical University Hospital, Taichung, 40403 Taiwan, ROC; 4 Research Center for Cancer Biology, China Medical University, Taichung, 40403 Taiwan, ROC; 5 Department of Medical Imaging and Radiological Sciences, Chang Gung University, Taoyuan, 33302 Taiwan, ROC; 6 Department of Radiation Oncology, Chang Gung Memorial Hospital at Linkou, Taoyuan, 33302 Taiwan, ROC; 7 Institute for Radiological Research, Chang Gung Memorial Hospital, Chang Gung University, Taoyuan, 33302 Taiwan, ROC; 8 The Ph.D. Program for Cancer Biology and Drug Discovery, China Medical University and Academia Sinica, Taichung, 40403 Taiwan, ROC; 9 Drug Development Center, China Medical University, Taichung, 40403 Taiwan, ROC; 10 Cancer Genome Research Center, Chang Gung Memorial Hospital at Linkou, Taoyuan, 33302 Taiwan, ROC; 11 Institute of New Drug Development, China Medical University, Taichung, 40403 Taiwan, ROC

**Keywords:** Cancer, Computational biology and bioinformatics, Systems biology

## Abstract

Cervical cancer is the fourth most common cancer in women worldwide. Increasing evidence has shown that miRNAs are related to the progression of cervical cancer. However, the mechanisms that affect the prognosis of cancer are still largely unknown. In the present study, we sought to identify miRNAs associated with poor prognosis of patient with cervical cancer, as well as the possible mechanisms regulated by them. The miRNA expression profiles and relevant clinical information of patients with cervical cancer were obtained from The Cancer Genome Atlas (TCGA). The selection of prognostic miRNAs was carried out through an integrated bioinformatics approach. The most effective miRNAs with synergistic and additive effects were selected for validation through in vitro experiments. Three miRNAs (miR-216b-5p, miR-585-5p, and miR-7641) were identified as exhibiting good performance in predicting poor prognosis through additive effects analysis. The functional enrichment analysis suggested that not only pathways traditionally involved in cancer but also immune system pathways might be important in regulating the outcome of the disease. Our findings demonstrated that a synergistic combination of three miRNAs may be associated, through their regulation of specific pathways, with very poor survival rates for patients with cervical cancer.

## Introduction

Cervical cancer is the fourth most common cancer in women worldwide and the most common cancer in some low-resource countries, with an estimated 570,000 cases and 311,000 deaths per year globally^1,2^. The most common histological type of cervical cancer is squamous cell carcinoma, which accounts for more cases than any other type^3^. The primary treatment for patients with cervical cancer include surgery, chemotherapy, and radiotherapy^4,5^. Despite the advances of modern medicine, approximately a quarter of patients with cervical cancer will experience cancer recurrence or death within 3 years^6^. Therefore, biomarkers that could be used to select those patients with poor prognosis in advance for intensified treatment are needed.

A microRNA (miRNA) is a small molecule RNA with the length of 22 nucleotides that can regulate the expression of oncogenes and tumor suppressor genes through the post-transcriptional level and, therefore, affect the progress of cancer^7,8^. Various miRNAs have been regarded as potential biomarkers for cancer development and as targets for cancer treatment^9–12^. Evidence from cell lines and malignant tissues have provided evidence for the role of miRNAs in the development of cervical cancer^13,14^. However, results showing how specific miRNAs affect the clinical outcomes of cervical cancer and the relevant mechanisms have been limited and inconsistent in previous studies^15,16^. To clarify the role of miRNAs in the clinical prognosis of cervical cancer, a large cohort including comprehensive miRNA sequencing data and reports of patient outcomes is needed. The Cancer Genome Atlas Project (TCGA), which the National Institutes of Health (NIH) launched in 2006, is one of the most comprehensive and largest gene sequencing projects. TCGA provides clinicopathologic annotation data along with multi-platform molecular profiles of more than 11,000 human tumors across 33 different cancer types^17^.

To date, limited studies have investigated the prognosis-related multiple miRNAs networks in cervical cancer with high-throughput data in databases such as TCGA database with big data analytic^15,16^. Gao et al.^15^ identified four miRNAs (miR-99a, miR-125b, miR-188, and miR-223) individually related to the survival of patients with cervical cancer by analyzing the GEO and TCGA databases. Liang et al. (2017)^16^ recognized a 3-miRNA signature (miRNA-145, miRNA-200c, and miRNA-218-1) predicting the survival of cervical cancer. However, these studies were bioinfomatics approches alone and lack the experimental validation. In the present study, we identified three survival related miRNAs by analyzing the miRNA and clinical data from TCGA with additive effect, and then validated these miRNAs through in vitro experiments. Furthermore, function enrichment analysis of the target genes of these three miRNAs showed that not only pathways traditionally involved in cancer but also immune system pathways play an important role in the prognosis of cervical cancer.

## Results

### Identification of the miRNA signature related to prognosis

**Figure 1:**
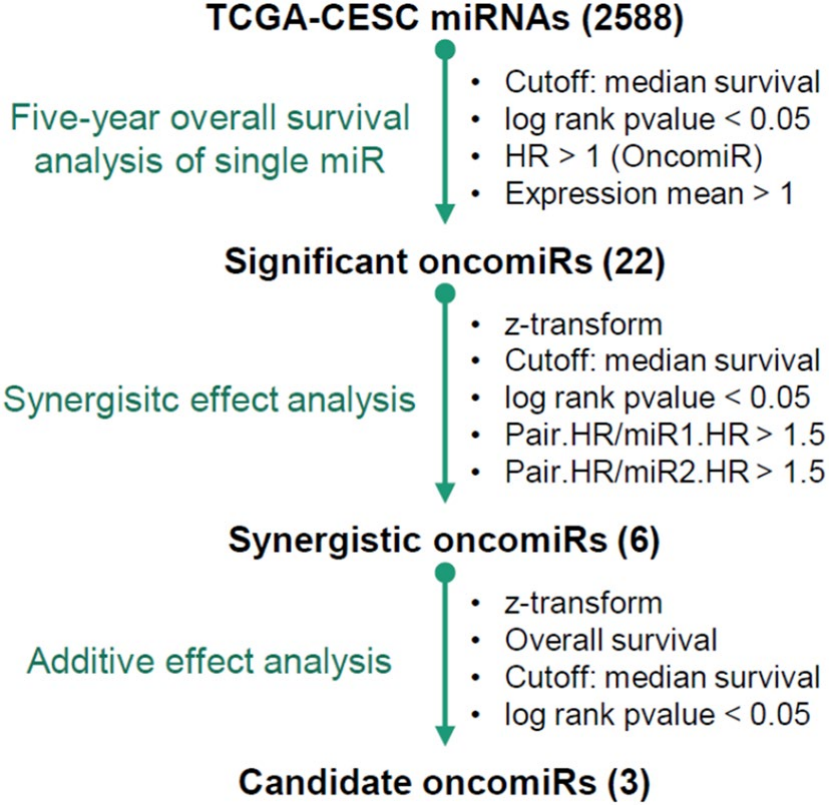
miRNA-seq analysis and prognostic miRNAs generation pipeline.

**Figure 2:**
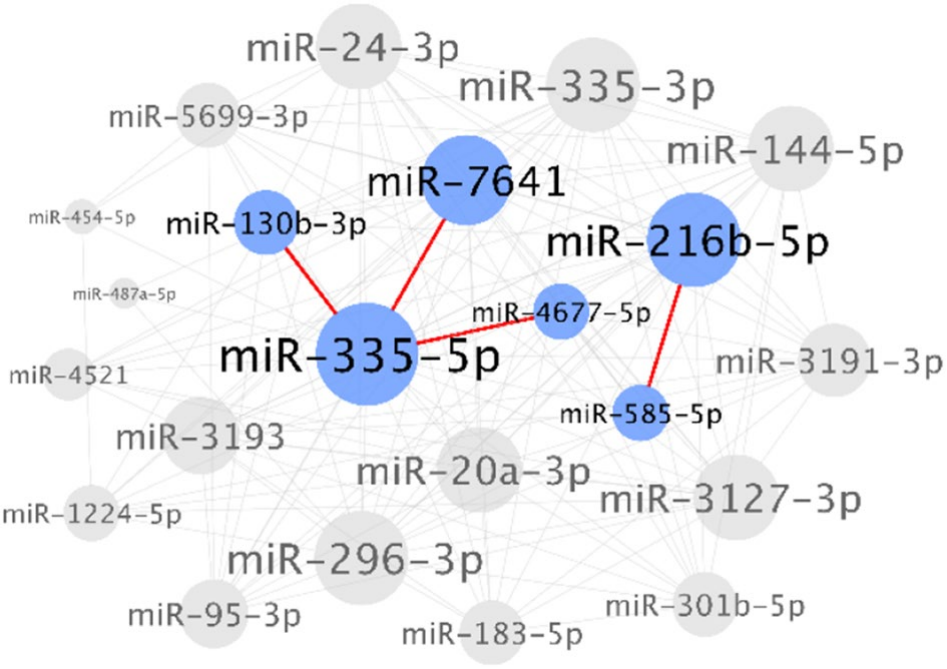
Synergistic survival analysis of 22 candidate miRNAs indicated that only 6 miRNAs (blue circles) had significantly synergistic effects when combined in 4 pairs (red lines).

**Figure 3:**
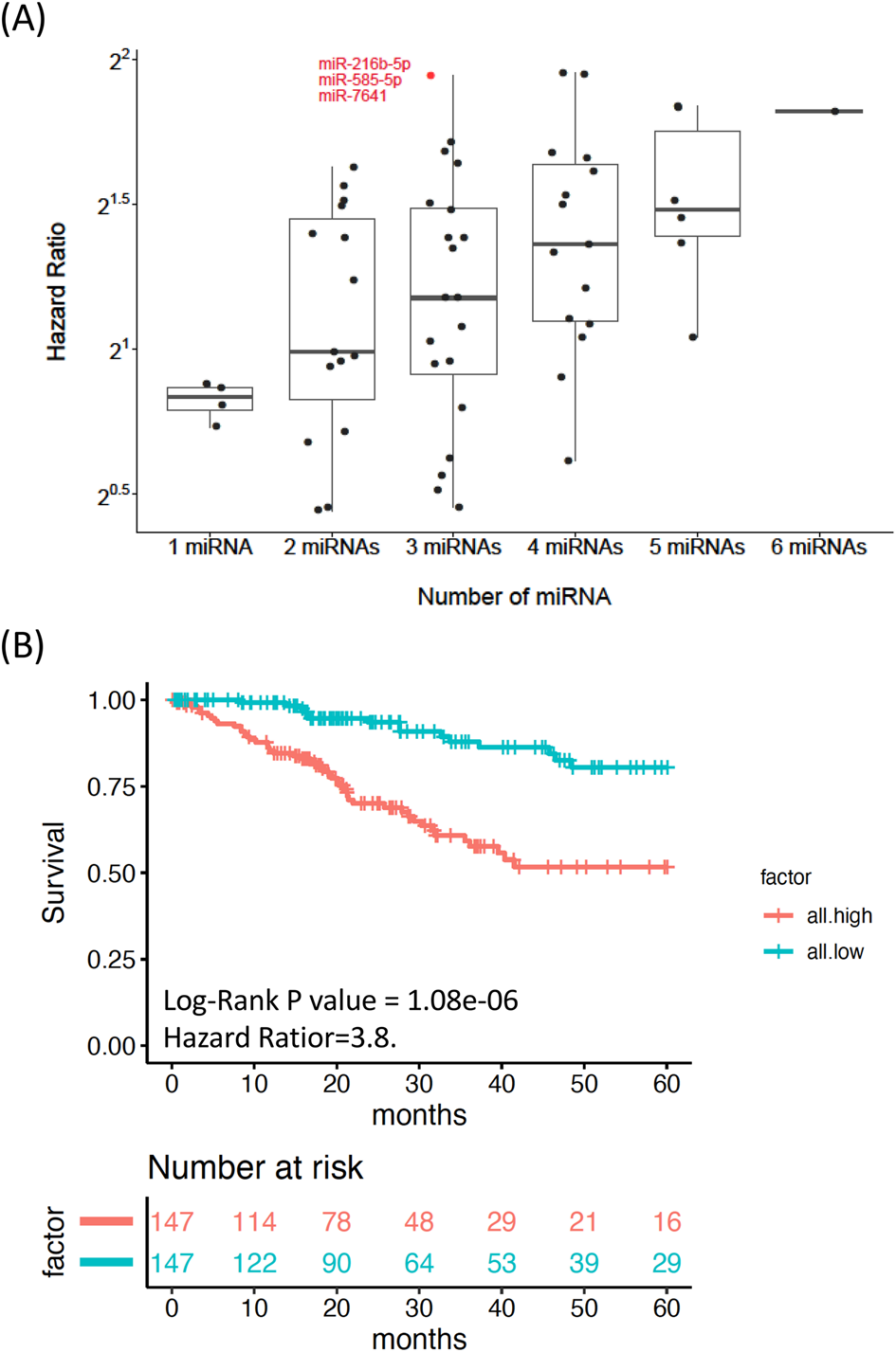
Additive survival analysis of 6 candidate miRNAs. **(A)** The hazard ratios (HR) of each of the miRNA combinations were calculated by additive survival analysis, as shown by the boxplots with dotplots overlaid. The X-axis indicates the number of miRNAs combined; the Y-axis indicates the log2 transformation of the hazard ratio. The red spot indicates the combination of miR-216b-5p, miR-585-5p, and miR-7641. **(B)** Survival analysis of the combination of the 3 miRNAs shows a significant difference between the all-high and all-low expression groups with log-rank p-value < 0.05.

MiRNA could either act as oncogenic or tumor suppressive. Generally, oncogenic miRNAs (oncomiRs) were overexpressed in cancer and played an important role in tumorigenesis^18^. In order to comprehensively analyze the prognostic oncomiRs of cervical cancer, we developed an integrated bioinformatics pipeline (Fig. 1). Firstly, The Cancer Genome Atlas Cervical Squamous Cell Carcinoma and Endocervical Adenocarcinoma (TCGA-CESC) data with the total of 294 tumor samples were enrolled, and a total of 2588 miRNAs were used for further investigation. 22 significant survival-related oncomiRs were identified according to the survival analysis (log-rank p-value < 0.05 and HR > 1). To clarify whether two genes with high-level co-expression resulted in very poor outcomes in terms of survival, we evaluated the synergistic effects between these 22 miRNAs according to the algorithm developed in our previous study^19^. Only six miRNAs (miR-130b-3p, miR-216b-5p, miR-335-5p, miR-585-5p, miR-4677-5p, and miR-7641) combined in four pairs showed significant synergistic effects (Fig. 2; Table 1). In order to determine the optimal combination of the potential prognosis-related miRNAs, we further examined the additive effects of survival in these six miRNA candidates (Fig. 3). In Fig. 3A, the hazard ratio (HR) of different miRNAs in combination were gradually increased when more miRNA candidates were included, which indicated that more of the miRNAs in combination contributed to poorer survival. For example, “3 miRNAs” in the X axis of the Fig. 3A indicates the all combinations of any three miRNAs and the best combination is consisted of miR-216b-5p, miR-585-5p, and miR-7641 (the red dot in Fig. 3A), presented with roughly the highest HR (3.85, log-rank p value = 1.08e−06) in Fig. 3A. The Kaplan–Meier survival analysis also demonstrated a significant difference between the all-high and all-low expression groups through the integration of these three miRNAs (Fig. 3B). Therefore, the three miRNAs and their integration were selected as our preferred candidates for further functional annotation and experimental validations.

**Table 1:** The hazard ratios of the significantly synergistic effects of 6 candidate miRNAs combined in 4 pairs.

miR1	miR2	p value (pair)	HR (pair)	HR (miR1)	HR (miR2)
miR-335-5p	miR-4677-5p	4.33E−05	3.09	1.75	1.84
miR-335-5p	miR-7641	1.45E−04	2.82	1.75	1.82
miR-216b-5p	miR-585-5p	6.58E−05	2.95	1.92	1.65
miR-130b-3p	miR-335-5p	3.13E−04	2.64	1.66	1.75

*HR* hazard ratio.

### Identification of the target genes of the miRNA signature

**Figure 4:**
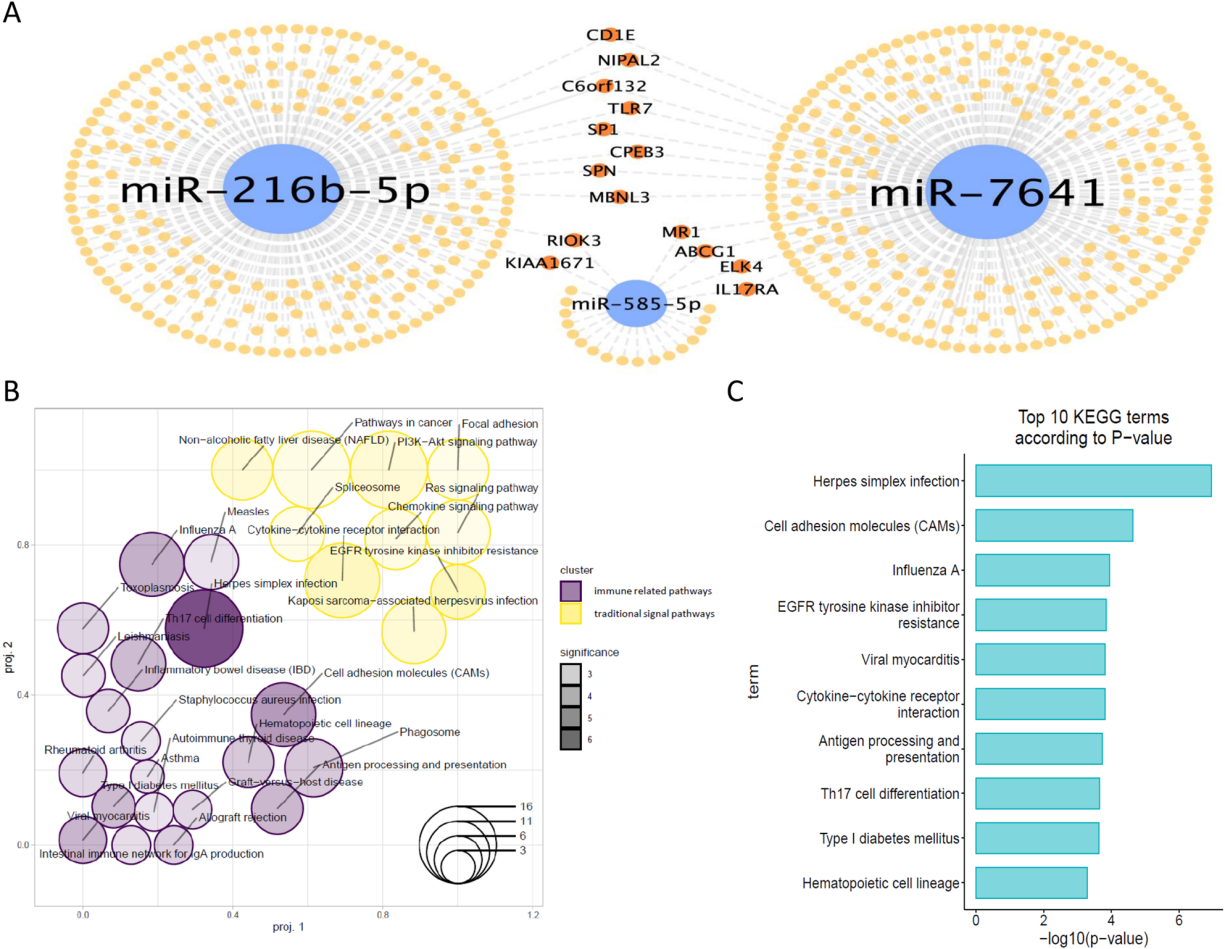
Target gene interaction and functional enrichment. **(A)** The collaborative network displaying the interactions between the 3 candidate miRNAs and the targeted genes. **(B)** A gene set overrepresentation of the top 30 significant KEGG pathways. **(C)** The top 10 significant enriched KEGG pathways of the target genes.

Furthermore, we also identified the 536 target genes of the three miRNAs (miR-216b-5p, miR-585-5p, and miR-7641) according to the following criteria: (1) negative correlation (the correlation coefficient between the miRNA and mRNA was < 0), (2) survival significance (the log rank p < 0.05), and (3) recorded in the miRTarbase or predicted by more than four miRNA target prediction tools. As illustrated in Fig. 4A, the network of miR-gene interactions showed that the overlapping genes regulated by the miRNAs were few in number, which indicated that the regulatory mechanisms involved might be different. Then, functional annotation was performed to elucidate the crucial functions regulated by the target genes. For the top 30 most significant functions of *Kyoto Encyclopedia of Genes and Genomes* (KEGG), we also utilized gene set over-representation analysis to illustrate that these functions could be divided into two main groups (Fig. 4B). One group represented traditional signal pathways, including the pathways involved in cancer, EGFR tyrosine kinase inhibitor pathways, PI3K-Akt signaling pathways, and Ras signaling pathways. Another group included functions relating to T cell differentiation, virus infection, and immune diseases, which suggested that the infection and immune system may also play an important role in the prognosis of patients with cervical cancer. Focusing on the top 10 KEGG terms indicated that EGFR tyrosine kinase inhibitor resistance and the adaptive immune system involving T cell differentiation against herpes virus infection might play the crucial role (Fig. 4C). The top 10 *Gene Ontology* (GO) and Reactome pathways also showed significant biological functions involved in the immune- and infection-related pathways (Supplementary Fig. 1A,B).

### In vitro assays for validation of the miRNA signature

**Figure 5:**
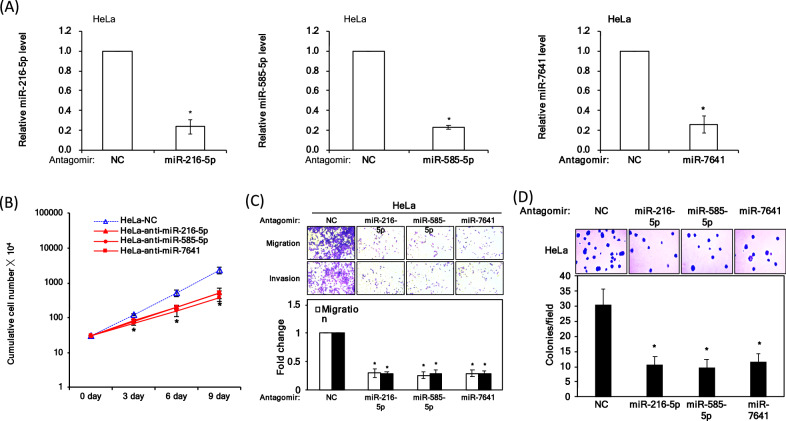
Overexpression of antagomiRs abolished the growth and tumor progression of HeLa cells in vitro. **(A)** AntagomiR qRT-PCR analysis results. **(B)** Cell growth assay results. **(C)** Soft-agar colony formation assay results. **(D)** Migration and invasion assay results.

To certify the miRNAs signature identified by the integrated bioinformatics approach, we utilized antagomiRs to knockdown the three miRNAs in HeLa cells (Fig. 5A) and performed the experimental validation of three antagomiRs in multiple functional assays. First of all, we checked the basal expression levels of the three miRNAs. The high threshold cycle (Ct) number of RNU48 (endogenous control), miR-216b-5p, miR-585-5p, and miR-7641 (23.5, 22.96, 23.08, and 15.5) indicated good expression of the three miRNAs in Hela cells. Cell growth assays indicated that the number of HeLa cells significantly decreased when transfecting with the antagomiRs compared to a control (Fig. 5B). Colony formation assays also indicated significant reductions after transfection with each of the antagomiRs (Fig. 5C). As shown in Fig. 5D, the cell migration and invasion assay results showed more than twofold declines after antagomiR transfection. Furthermore, the cell growth, migration, and invasion assay all showed more significant decrease with three miRNAs combined antogomiRs by using equal final concentrations. (Supplementary Fig. 2A,B). Taken together, these experimental validations demonstrated that the three miRNAs are related to cancer progression and severity.

## Discussion

MiRNAs have been well known to regulate the different biological pathways by altering gene expression, which in turn affects the growth of various types of tumors, including cervical cancer ^20^. To date, several miRNAs that may be related to the clinical prognosis of cervical cancer have been reported, such as miR-31^21^, miR-155^22^, miR425-5p^23^, miR638^24^, miR-944^25^, and miR-1254^26^. However, these heterogeneous results may have been generated by studies with small sample sizes, studies with relatively limited numbers of candidate miRNAs, those considering only single markers, or those lacking experimental validation. In our study, we identified three cervical cancer-prognosis-related miRNAs (miR-216b-5p, miR-585-5p, and miR-7641) through an integrated bioinformatics approach and then validated these three miRs through multiple functional assays. Of the three miRNAs included in the miRNA signature, miR-216b-5p has been reported to be associated with the progression of several types of cancer, including breast cancer^27^ , liver cancer^28^, pancreatic cancer^29^, and cervical cancer^30^. He et al. (2017) reported that miR-216b altered the prognosis of cervical cancer by inhibiting cell proliferation through target FOXM1 in cervical cancer cells^30^. MiR-585-5p has been found to suppress tumor proliferation in gastric cancer and non-small cell cancer by targeting MAPK1 and hSMG-1^31,32^. miR-7641 has been found to be a potential marker for gastric cancer^33,34^ and might be a therapeutic target in cancer therapy^35^. These previous results suggested that our candidate miRNAs might be clinically important cancer biomarkers. The inhibition of these three miRNAs could thus potentially improve the outcomes of patients with cervical cancer.

To more fully understand the molecular functions of these three miRNAs, we identified the target genes of the three miRNAs through the miRTarBase database and bioinformatics tools for miRNA target prediction. Moreover, we performed functional enrichment analysis of those target genes based on the GO, KEGG, and REACTOME databases. Interestingly, the infection and immune system seemed to play major roles according to the REACTOME analysis, and this system was also identified as important through several pathways in the GO and KEGG databases. The immune system has close relationships with cancer initiation, progression, and metastasis. In cervical cancer, immune defenses against viral infection are important for eliminating the risk of cancer initiation, which indicates the benefits of vaccines to prevent cancer development in high-risk people^36^. For many cancers, immunotherapy, which refers to treatments used to treat cancer involving components of the immune system, has become clinically validated in recent years^37^. To date, however, only two phase II trials have validated the benefits of immunotherapy for cervical cancer. Specifically, the Keynote 158^37^ and Checkmate 358^37^ studies demonstrated that immune checkpoint inhibitors such as nivolumab and pembrolizumab may help against advanced and recurrent cervical cancer. However, the objective response rates (ORRs) thus far have been low, ranging from 12.2 to 26.3%. Therefore, combinations of other therapies to improve response rates have been attracting considerable attention. Several microRNAs have been noted as target checkpoint molecules that can mimic the therapeutic effects of a combined immune checkpoint blockade^38^. Relatedly, the results of our study might be helpful in terms of selecting the three specific miRNAs, which have the potential to provide new targeted therapies for cervical cancer with or without current immunotherapies.

To date, only a few studies have investigated prognosis-related multiple miRNA networks in cervical cancer, with those studies using high-throughput data with bioinformatics approaches alone^15,16^. Gao et al. (2018)^15^ obtained eight up-regulated miRNAs and 13 down-regulated differential miRNAs by first analyzing the data of gene expression microarray (GSE30656) from the GEO database, and then identifying four miRNAs that have the highest predictive valus and are associated with patient survival using Cox regression analysis based the data in the TCGA database. Those four miRNAs, namely, miR-99a, miR-125b, miR-188, and miR-223, may regulate several signaling pathways, including the mTOR signaling pathway, signaling pathways regulating the pluripotency of stem cells, and the MAPK signaling pathway, as well as microRNAs in cancer. On the other hand, by analyzing only TCGA data, a total of 78 differentially expressed miRNAs were recognized, and three survival predicting miRNA (miRNA-145, miRNA-200c, and miRNA-218-1) were identified through Cox regression analysis by Liang et al. (2017)^16^. The functional enrichment analysis indicated that various pathways, including the MAPK, AMPK, focal adhesion, cGMP-PKG, wnt, and mTOR signaling pathways, were regulated by these miRNAs. Interestingly, the crucial role of the infection and immune system was only noted in our study. This discrepancy might be contributed by the different prognosis related miRNAs (miR-216b-5p, miR-585-5p, and miR-7641) we identified. In our study, instead of filtering miRNAs through differential expression, we focused only on survival related oncomiRs and examined them using synergistic and additive analyses. The analyses of additive oncomiRs could help us to recognize the most impactful miRNA signature related to the progression of cervical cancer and the potential therapeutic target^39^. In comparison to these previous two studies, our study performed experimental studies to validate the bioinformatics results.

In conclusion, our study identified a novel three-miRNA signature which could be a candidate biomarker for the prediction of poor cervical cancer prognosis. Our findings also demonstrated that the immune system may play an important role in this complex miRNA-gene network. Finally, large prospective cohort studies may be required for the further validation, and additional experimental studies in vivo are also needed to provide robust evidence of the significant role of the miRNAs in tumor progression.

## Materials and methods

### Data processing

The miRNA sequencing data and clinical information of TCGA- CESC dataset were obtained from our previous studies and the YM500 and DriverDB databases^19,40–44^. In brief, the miRNA-seq, RNA-seq, and clinical data of TCGA-CESC were obtained from Genomic Data Commons (GDC, https://gdc.cancer.gov/). As such, 294 tumor samples of miRNA-seq data were included for the following analyses. The data preparation and processes for the miRNA-seq and RNA-seq data were addressed using in-house Perl and R language scripts, the details of which are documented in previous publications^40,43^. The detailed clinical characteristics of the included patient samples are listed in Table 2.

**Table 2:** Basic characteristics of TCGA-CESC patients.

Variables	Case, n (%)
**Age**	
≥ 60	61 (20.8%)
< 60	232 (78.9%)
N/A	1 (0.3%)
**Stage**	
I	160 (54.4%)
II	65 (22.1%)
III	42 (14.3%)
IV	21 (7.1%)
N/A	6 (2%)
**T stage**	
T1 + T2	206 (70.1%)
T3 + T4	27 (9.2%)
Others (T0 or Tis or Tx or N/A)	61 (20.7%)
**N stage**	
N1	55 (18.7%)
Others (No or Nx or N/A)	239 (91.3%)
**M stage**	
M1	10 (3.4%)
Others (M0 or Mx or N/A)	284 (96.6%)

### Identification of survival-related miRNAs with synergistic and additive effects

The R *survival* package (version 2.41-3) was used to a calculate cox regression (or cox proportional hazards) model between two pre-defined groups. Patients with cervical cancer were stratified by the median of miRNAs expression. Each significant survival-relevant miRNA was identified with a log-rank p-value < 0.05 and a HR > 1. Also, to filter out the candidates with extremely low expression levels, we further selected those with a mean of normalized counts (reads per million, RPM) > 1. We also utilized the synergistic effect developed in our previous studies^19^ to identify the miRNA pairs with an HR value between two survival-relevant miRNAs whose combined expression was > 1.5 fold of each miRNA with a log-rank P-value < 0.05. Then, we constructed an analytic model to evaluate the additive effects among those miRNAs with synergistic effects. First, we calculated all of the outcomes of any combinations of miRNA candidates. After using the combinations of different miRNAs and the survival criteria above, we stratified the patients by the medians of miRNA expression levels within tumors. For each miRNA combination, the patients were classified into an all-high or all-low groups; the all-high group consisted of the patients who had very high expression of all the miRNAs in the combination, and vice versa. Then, survival estimations were computed based on the patients in the all-high and all-low groups. Significant combinations were defined with log-rank p-values < 0.05 and HR values > 1.

### Target gene prediction and functional annotation

The target genes of the prognostic miRNAs were predicted using the following two steps. First, the correlations between the RNA expression and miRNA expression levels were calculated, including three types of correlation: Pearson, Spearman, and Kendall correlations. Those genes and miRNAs for which one of the three types of correlations was < − 0.3 were taken into account. Second, to further enhance the bioinformatics analysis reliability, the miRTarbase (http://mirtarbase.mbc.nctu.edu.tw/php/index.php) data was downloaded and applied to show the interactions as networks^45^. Not only were the interactions recorded in the miRTarbase used but 12 prediction tools were applied to investigate the relations between the miRNA candidates and target genes, as detailed in our previous publication^41^. The interactions that were predicted by more than four tools were included for further analysis.

Functional enrichment analysis of the target genes was also performed as detailed in our previous publications^19,43,44^, with GO, KEGG, and Reactome pathway analyses all being applied. The adjusted p-value < 0.05 was set as the cut-off criterion. Gene set over-representation analysis was performed using the R package GSOAP^46^, which explores overlapped gene sets in multiple functions in top 30 significant KEGG terms. The top 10 significant GO, KEGG, and Reactome terms were also extracted from the annotation results and regarded as the most critical functions regulated by the miRNA candidates.

### Cell culture and miRNA transfection

The human cervical cancer line HeLa was obtained from and authenticated by the Bioresource Collection and Research Center (*BCRC*, Hsinchu, Taiwan). Cells were cultured in Dulbecco's Modified Eagle Medium (DMEM) supplemented with 10% fetal bovine serum (FBS) and 1% penicillin/streptomycin (PS; Gibco), and maintained at 37 °C in a humidified incubator containing 5% CO_2_. Hela cells were transfected with antagomirs (antogomiR-216-5p, antogomiR-585-5p, and antogomiR-7641) or a scrambled oligonucleotide as a negative control (Ambion) at a final concentration of 20 nM using Lipofectamine 2000 (Invitrogen) according to the manufacturer’s instructions. For cell growth assay, equal numbers (approximately 3 × 10^5^) of the transfected cells were seeded into six-well plates and then counted using the trypan blue exclusion method at the indicated times.

### Quantitative real-time PCR

Total RNA was extracted using the TRIzol reagent (Invitrogen) according to the manufacturer’s instructions. To detect the level of mature miRNAs, t total RNA was reverse transcribed into complementary DNA using the MultiScribe Reverse Transcriptase system and the specific primers designed for miR-216-5p, miR-585-5p, or miR-7641 (Applied Biosystems). Real-time PCR were performed using TaqMan Universal PCR Master Mix and TaqMan MicroRNA Assays (Applied Biosystems) in a ABI StepOne Plus system. The relative expression level of each miRNA was compared to the RNU48 endogenous control and normalized to cells transfected with scramble miRNA (NC group) using the 2 − ΔΔCt method.

### Colony formation, migration, and invasion assays

For the formation of colonies, equal numbers of the transfected cells were used for the assay of anchorage-independent growth in soft agar and cultured for 14 days. Then, the cells were stained with crystal violet, and the colonies were counted from ten random fields under a microscope^47,48^. The migration and invasion ability assays were performed in 24-well plates (1 × 10^4^ cells for the migration assay and 5 × 10^4^ cells for the invasion assay) using Millicell tissue culture insert well plates (Millipore) for 12 h and BD BioCoat Matrigel Invasion Chambers (Becton Dickson) for 20 h, respectively^48,49^.

### Statistical analysis

Statistical analyses for biological experiment were performed using the Student’s *t*-test for a simple comparison of the two groups. Differences were considered statistically significant if the *P* value was < 0.05.

## Supplementary Information

Supplementary Figures.

## Abbreviations

TCGA: The cancer genome atlas
CESC: Cervical squamous cell carcinoma and endocervical adenocarcinoma
GO: Gene ontology
KEGG: Kyoto encyclopedia of genes and genomes
