# Supplementary material for: Identification and validation of a miRNA-based prognostic signature for cervical cancer through an integrated bioinformatics approach
Source: Sci Rep. 2020 Dec 17;10:22270. doi: 10.1038/s41598-020-79337-4 (PMC7747620; doi:10.1038/s41598-020-79337-4)
Supplement: Supplementary file 1 — Supplementary Figures. [file 41598_2020_79337_MOESM1_ESM.pdf]

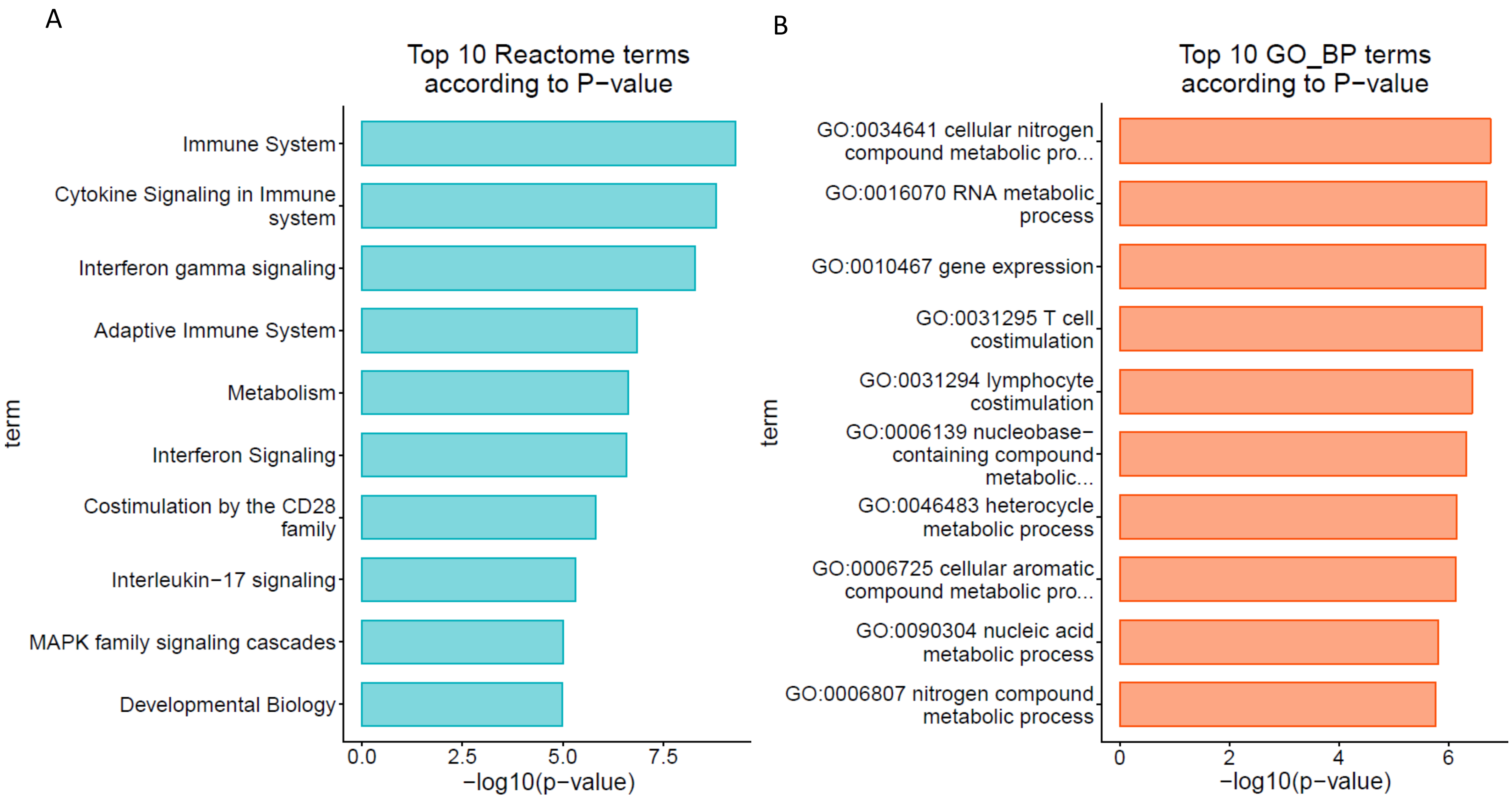

Figure S1. Functional enrichment analysis of target genes of the three miRNAs by Reactome (A) and GO (B)

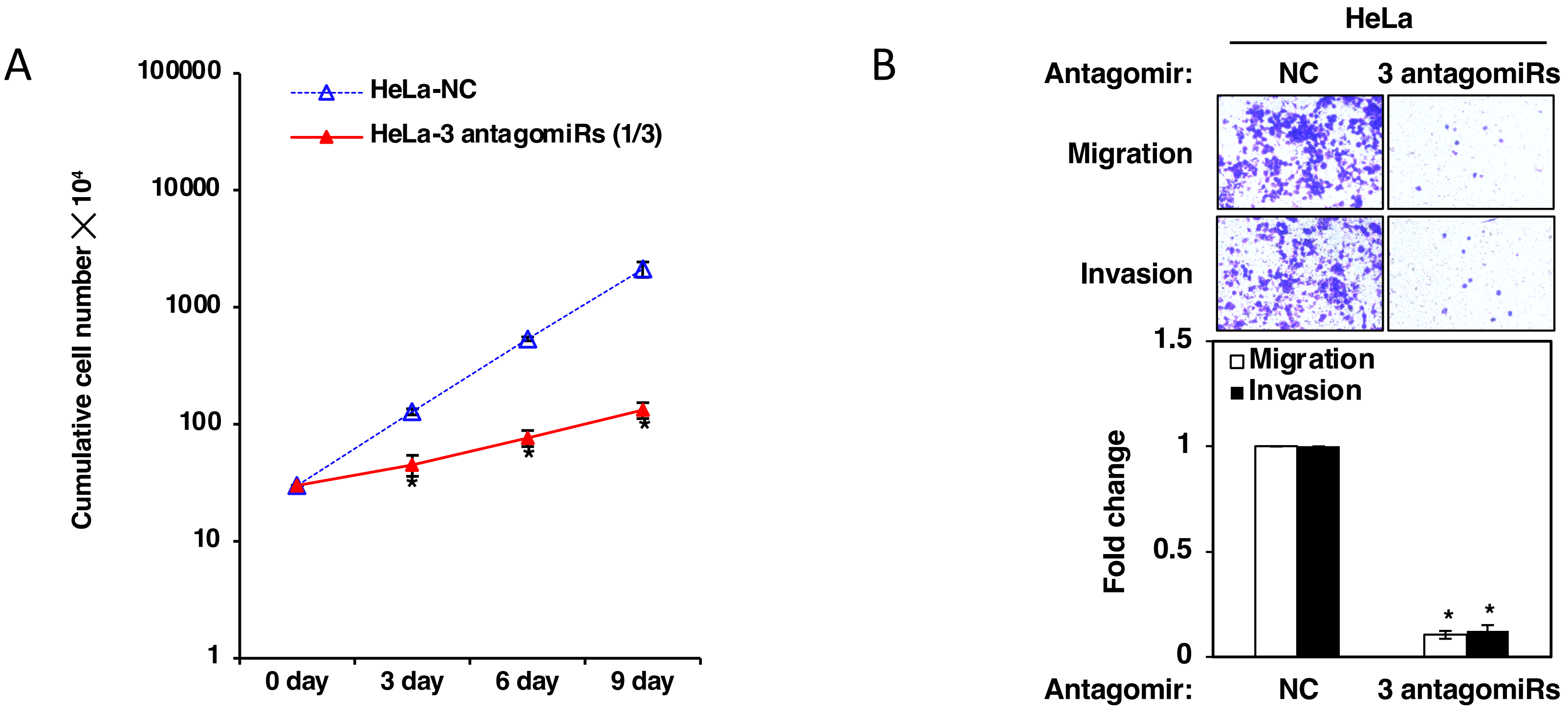

Figure S2. Knockdown the 3 miRNA in Hela cell line. (A) proliferation assay. (B) migration and invasion assays
